# GO@CNT@Fe₃O₄@CuO quaternary nanohybrids enhance dielectric-magnetic synergy for high-performance epoxy-based electromagnetic absorbers

**DOI:** 10.1038/s41598-026-41828-1

**Published:** 2026-02-26

**Authors:** Leila Akbarzadeh Gholidizchi, Morad Ebrahimkhas, Hossein Hooshyar

**Affiliations:** 1 Department of Physics, MAH.C, Islamic Azad University, Mahabad, Iran; 2 Department of Chemistry, MAH.C, Islamic Azad University, Mahabad, Iran

**Keywords:** GO@CNT@Fe_3_O_4_@CuO nanohybrid, Electromagnetic wave absorber materials, Epoxy resin, Nanocomposite, Core-shell structure, Engineering, Materials science, Nanoscience and technology

## Abstract

This study introduces a novel quaternary GO@CNT@Fe₃O₄@CuO core-shell nanohybrid designed to overcome impedance mismatch limitations in electromagnetic (EM) absorbers. Through stepwise synthesis, we integrated dielectric (GO, CNT), magnetic (Fe₃O₄), and semiconducting (CuO) components to create synergistic loss mechanisms. When incorporated at only 5 wt% into epoxy resin, the nanocomposite achieved exceptional X-band absorption with a minimum reflection loss of − 37.5 dB at 10.25 GHz and an effective absorption bandwidth of 3.2 GHz (9.0–12.2 GHz) at 5.0 mm thickness. The enhanced performance stems from balanced complex permittivity (ε′ = 6.1, ε″ = 2.6) and permeability (µ′ = 1.28, µ″ = 0.19), enabling optimal impedance matching and multi-mechanism attenuation through conduction loss, interfacial polarization, and magnetic resonance. This work establishes a design principle for low-loading, high-efficiency EM absorbers suitable for 5G and aerospace applications.

## Introduction

Recently, the rapid progress of modern technologies has become clear. Indeed, the deployment of 5G networks has significantly enhanced data transmission speeds and connectivity, enabling advanced applications in telecommunications, healthcare, and smart infrastructure^1^. Almost every application, including the biomedical field, radar systems, communication networks, and more, is widely based on electromagnetic (EM) waves^2^. Although these EM wave applications are intriguing, they face considerable challenges stemming from overuse. As technology advances, the popularity of multifunctional wearable electronics encompassing health tracking^3^, motion sensing^4^, thermal regulation^5^, and more steadily increases. While these features enhance our comfort, they also pose risks to human health and the environment due to EM waves’ heating and non-heating impacts. EM pollution is increasing daily, posing a significant problem that requires urgent action. EM waves disrupt communication networks and electronic systems, resulting in obstacles to effective communication and the reliable operation of these systems. Furthermore, these EM radiations impact human health, contributing to the development of cancer cells, compromising immunity, and modifying the DNA structure^6^. Consequently, creating innovative, efficient electromagnetic microwave absorbing materials (MAMs) has emerged as a key area of research to mitigate the detrimental effects of EM waves. Creating a multifunctional EM absorber for EM shielding is challenging, as a single material typically cannot possess multiple characteristics, complicating the task significantly. Nevertheless, this issue can be addressed by customizing the material design into a multi-layer structure at the nano or micro level, which leads to multifunctional properties. This is currently an active research area in advancing EM wave absorbers^7,8^.

Carbon allotropes are widely used in electromagnetic wave absorbers due to their advantageous properties, including low density, excellent electrical conductivity, low thermal expansion, high thermal conductivity, and corrosion resistance^9,10^. Their effectiveness mainly comes from dielectric loss, where carbon acts as a resistive element that converts incident microwave energy into heat through Joule heating. The strong response of electric dipoles within carbon structures to the alternating electric field of electromagnetic waves explains their superior ability to absorb microwaves. Similarly, magnetic loss materials reduce the magnetic field component of microwaves. Magnetite (Fe_3_O_4_) is a particularly attractive magnetic filler for enhancing microwave attenuation in carbon-based composites (e.g., carbon nanotubes) owing to its low toxicity, good compatibility, high spin polarization, and significant room-temperature magnetic properties^11–13^. Incorporating metal oxide hybrids with magnetite further improves absorption characteristics. These hybrids facilitate key loss mechanisms within the composite, including conduction loss, residual losses, interfacial polarization, electron spin resonance, and resonant domain wall motion^14,15^.

Consequently, a highly effective strategy involves embedding hybrid nanoparticles—such as magnetite/metallic or magnetite/semiconductor systems—into conductive polymers or onto carbon substrates (notably carbon nanotubes). This approach yields composites with strong, broadband microwave absorption performance. Following this principle, the narrow-bandgap semiconductor copper oxide (CuO) has been successfully utilized to decorate various carbon materials (e.g., carbon fibers, carbon black, carbon nanotubes, graphene) to create high-performance microwave-absorbing composites^16^. Therefore, many researchers have used CuO nanoparticles to improve the EM wave absorption properties. Hekmatara et al. developed new quaternary MWCNT/CuO/Fe_3_O_4_/PANI nanocomposites with different weight ratios of CuO/Fe_3_O_4_/PANI to MWCNT (1:3, 1:4, and 1:5). They used TEM and SEM to characterize these nanocomposites. All demonstrated excellent microwave absorption, with the (1:5) ratio achieving the highest reflection loss at − 87.4 dB, and the (1:4) ratio offering the widest absorption bandwidth at 7.6 GHz (RL ≤ − 10 dB). These findings underscore their potential for advanced electromagnetic shielding applications^17^. While a wide variety of polymer and inorganic matrices—including polystyrene (PS), polyurethane (PU), polyvinylidene fluoride (PVDF), silicone rubber, hydrogels, gypsum, and even brick—have been explored as hosts for microwave-absorbing fillers^18–22^, epoxy resin stands out as an optimal matrix for high-performance, structurally robust absorbers. Unlike PS or polyethylene (ε′ ≈ 2.0–2.5), which offer poor filler–matrix interaction and insufficient polarization, or PVDF (ε′ > 8), which often causes severe impedance mismatch due to excessive permittivity, epoxy provides a balanced dielectric constant (ε′ ≈ 3.0–4.0) that facilitates superior impedance matching when combined with conductive/magnetic nanofillers. Moreover, the abundant epoxy and hydroxyl groups in the cured network enable strong electrostatic and hydrogen-bonding interactions with oxygen-functionalized GO, Fe₃O₄, and CuO surfaces. These interactions not only enhance nanofiller dispersion—suppressing agglomeration—but also create additional interfacial dipoles that contribute to enhanced interfacial (Maxwell–Wagner–Sillars) polarization, a critical loss mechanism in the X-band. Additionally, epoxy’s excellent mechanical strength, thermal stability (> 200 °C), chemical resistance, and adhesion make it uniquely suited for real-world applications in aerospace, defense, and 5G infrastructure, where durability under harsh conditions is essential—unlike soft matrices such as hydrogels or silicone rubber^18,21^. Thus, the strategic selection of epoxy is not merely a processing convenience but a key design element that actively regulates electromagnetic performance through tunable filler–matrix coupling^20,22^. Similar epoxy-based composites with hexaferrites have demonstrated enhanced radar absorption through structural design optimizations^23^ and zinc substitutions^25^, while doped polyaniline-epoxy systems highlight thermal-microwave synergies for stealth uses^24^. U-type barium hexaferrite-epoxy composites further illustrate tunable electromagnetic properties for broadband absorption^26^.

This study focuses on the X-band (8–12.5 GHz), which is critical for radar and satellite communication, though future work will extend to Ku and K bands and introduces a novel quaternary GO@CNT@ Fe_3_O_4_@CuO nanohybrid, produced through a step-by-step process for the first time, to enhance the electromagnetic (EM) absorption qualities of modified epoxy resin. The quaternary design enables superior impedance matching by balancing ε_r_ and µ_r_ through complementary contributions: GO and CNT moderate conductivity to avoid over-reflection, Fe₃O₄ provides magnetic permeability, and CuO fine-tunes dielectric response via defect polarization. This balance reduces reflection at the air-absorber interface, allowing deeper EM wave penetration compared to highly conductive CNT-only or purely dielectric systems. Initially, GO@CNT@ Fe_3_O_4_ hybrid nanoparticles were synthesized using an optimized protocol to achieve targeted magnetic properties and appropriate size distribution. Next, CuO nanoparticles were deposited onto the surface of the GO@CNT@Fe_3_O_4_ hybrid via an in situ method, producing the final GO@CNT@ Fe_3_O_4_@CuO nanohybrid (Copper oxide (CuO) was selected due to its narrow bandgap (~ 1.2–1.7 eV), which promotes defect-induced polarization and interfacial charge transfer. Unlike wide-bandgap oxides (e.g., ZnO, TiO₂), CuO exhibits higher electrical conductivity and stronger dipole interactions, enhancing dielectric loss. Additionally, CuO forms stable interfaces with Fe₃O₄, potentially enabling p-n junction effects that improve impedance matching and multiple scattering). The nanohybrid was characterized through various techniques: X-ray diffraction (XRD) identified crystalline phases, and Fourier transform infrared (FTIR) spectroscopy verified successful synthesis. The morphology of all nanomaterials was examined with scanning electron microscopy (SEM) and transmission electron microscopy (TEM), which confirmed the core-shell structure with GO@CNT as the core (diameter ~ 10–30 nm), Fe₃O₄ shell (~ 5–10 nm thick), and outer CuO layer (~ 2–5 nm thick) through distinct contrast layers.

These nanofillers, including the nanohybrid, were then embedded into an epoxy resin matrix to produce high-performance nanocomposites. The electromagnetic properties of the composites were measured over the frequency range of 8–12.5 GHz using a vector network analyzer. Reflection loss versus frequency curves were plotted for each sample at various thicknesses using the obtained data. We expect that the introduction of the CuO nanoparticles in the GO@CNT@Fe_3_O_4_@CuO final nanofiller plays a key role in improving the EM efficiency of the epoxy-based nanocomposite. In the current investigation, three key innovations to achieve exceptional performance: (1) Multi-scale interfaces that boost polarization loss are created by integrating conductive carbon networks (GO/CNT), magnetic Fe_3_O_4_ nanoparticles, and semiconducting CuO into a core-shell nanocomposite. (2) This approach ensures optimal dispersion and interfacial interactions, outperforming previous ternary hybrids. (3) The findings demonstrate highly competitive absorption efficiency in the X-band, exceeding most existing carbon-based absorbers. Unlike earlier studies focusing on binary or ternary systems, our design uniquely addresses the impedance matching problem by utilizing the complementary roles of each component: GO/CNT for conductive loss, Fe_3_O_4_ for magnetic resonance, and CuO for defect-related polarization. This method sets a new benchmark for creating highly efficient, multi-mechanism absorbers. The results demonstrate competitive absorption performance in the X-band, with an RL of −37.5 dB at only 5 wt% loading, surpassing many carbon-based absorbers such as RGO/Fe₃O₄ (−25 dB) and GO@CNT@Fe₃O₄ (−22 dB) at similar thicknesses. This approach not only optimizes each component’s function but also provides a scalable blueprint for developing advanced materials suitable for 5G, aerospace, and wearable electronics.

## Experimental

### Materials

GO nanoplates were synthesized Graphite flakes (Alfa Aesar, 325 mesh, 44 μm) were used. Sonication was performed in an ultrasonic bath (40 kHz, 300 W) for 1 h during oxidation, hydrochloric acid (HCl, 37.0%, Merck), sulfuric acid (H_2_SO_4_, 98%, Merck), hydrogen peroxide (H_2_O_2_, 30.0%, Merck), potassium permanganate (KMnO_4_, Merck), and phosphoric acid (H_3_PO_4_, 85%, Merck). Additionally, Multi-walled CNTs (Sigma-Aldrich, catalog #MWCNT-15, OD: 10–15 nm, length: 10–30 μm, purity > 95%) were used. To synthesize the Fe_3_O_4_ nanoparticles on the GO@CNT, DI water, ammonia solution (25.0 wt%), and iron salts, including ferrous chloride tetrahydrate (FeCl_2_·4H_2_O) and ferric chloride hexahydrate (FeCl_3_·6H_2_O), were purchased from Merck. Copper (II) acetate (Cu(CH3COO)_2_, Merck), N,N-Dimethylformamide (DMF, Merck) and sodium hydroxide (NaOH, Merck) were used in the in-situ synthesis of the CuO nanoparticles on the GO@CNT@Fe3O4 nanocomposite. The epoxy resin known as Bisphenol F (Epon 862) and the amine curing agent known as 2, 4-diethyl-6-methylbenzene-1, 3-diamine (EK 3402) were provided by Hexion company (USA) and employed to fabricate the epoxy-based nanocomposite.

### GO synthesis

The modified Hummers method synthesized highly oxidized and well-dispersed GO nanoplates^27^. In this synthesis, 360 mL of H_2_SO_4_ and 40 mL of H_3_PO_4_ were mixed in a glass container in an ice bath. Next, 3.0 g of graphite powder was added to the acid mixture under agitation, followed by 18.0 g of KMnO_4_ as an oxidizing agent over 3 h while maintaining temperature control (below 10 °C). The yellow-green suspension was stirred at 40 °C for 12 h. Afterward, the suspension was poured into DI-water (400 mL) at 0 °C and stirred until it reached room temperature. Then, 5.0 mL of H_2_O_2_ was slowly added to the suspension, which turned orange-yellow upon stirring. To separate the obtained GO, gel from the suspension, it was allowed to sit for 10 h. In the next step, the separated GO gel was added to 200 mL of HCl (10.0%) and stirred at room temperature for 8 h to remove any MnO_2_ formed on the GO nanoplates. The GO suspension was centrifuged at 10,000 rpm and washed with ethanol and DI water several times to obtain a neutral GO gel. Finally, the purified GO gel was dried in a freeze dryer for 24 h to produce GO powder.

### GO@CNT synthesis

The GO@CNT nanohybrid was synthesized using dispersion and mixing techniques. First, 1.5 g of the prepared GO was combined with 200 mL of DI water and sonicated in an ultrasonic bath to form a yellow-brown suspension. Next, 3 g of CNTs and 300 mL of NMP were placed in a homogenizer and stirred vigorously for one hour. The aqueous GO was then added and stirred for an additional 30 min. The final GO@CNT product was separated from the reaction mixture, washed with DI water, and dried in a vacuum oven at 60 °C for 6 h.

### GO@CNT@Fe_3_O_4_ synthesis

The GO@CNT@Fe_3_O_4_ nanostructure was prepared using the in-situ synthesis of Fe_3_O_4_ on the GO@CNT nanohybrid. First, 1.5 g of GO@CNT was added to 500 mL of DI water in a double-necked flask, and it was then placed into an ultrasonication bath to obtain a uniform GO@CNT suspension. To form the Fe_3_O_4_ nanoparticles on the GO@CNT nanohybrid, The Fe²⁺/Fe³⁺ molar ratio was maintained at 0.5 (12.3 g FeCl₂·4 H₂O and 21.2 g FeCl₃·6 H₂O) to ensure stoichiometric magnetite formation. Under nitrogen protection, 60 mL of Ammonia was added dropwise over 1 h to control nucleation rate and prevent rapid precipitation, ensuring uniform particle size, and then the in-situ formation of Fe_3_O_4_ nanoparticles continued at 65 °C for 5 h. After the reaction, the suspension was exposed to a magnetic field to separate the GO@CNT@Fe_3_O_4_. During purification, the GO@CNT@Fe_3_O_4_ nanostructure was washed several times with DI water to reach a neutral condition. Finally, the separated GO@CNT@Fe_3_O_4_ nanostructure was dried in a freeze dryer.

### Synthesis of GO@CNT@CuO

GO@CNT nanostructures were coated with CuO nanoparticles. Briefly, 1.5 g of GO@CNT nanohybrid particles were ultrasonically dispersed in 400 mL of DI water to ensure even distribution. This suspension was then transferred to a three-necked flask, where 100 mL of DMF was added while stirring at 1000 rpm. When the suspension reached 85 °C, a 0.2 M Cu(CH₃COO)₂ solution (prepared by dissolving 16 g Cu(CH₃COO)₂ in DI water) was added, followed by dropwise addition of 1 M NaOH (20 mL) to facilitate hydrolysis and precipitation of Cu(OH)₂, which dehydrates to CuO upon heating. DMF serves as a co-solvent for better dispersion, while NaOH acts as the base. The mixture was heated to promote in situ CuO formation. After heating in an oil bath at 90 °C for an hour, the mixture was cooled down. The resulting GO@CNT@CuO was separated by centrifugation, washed multiple times with DI water and methanol, then autoclaved at 180 °C for 12 h. Finally, the nanostructure was freeze-dried.

### Fabrication of GO@CNT@Fe_3_O_4_@CuO

**Fig. 1:**
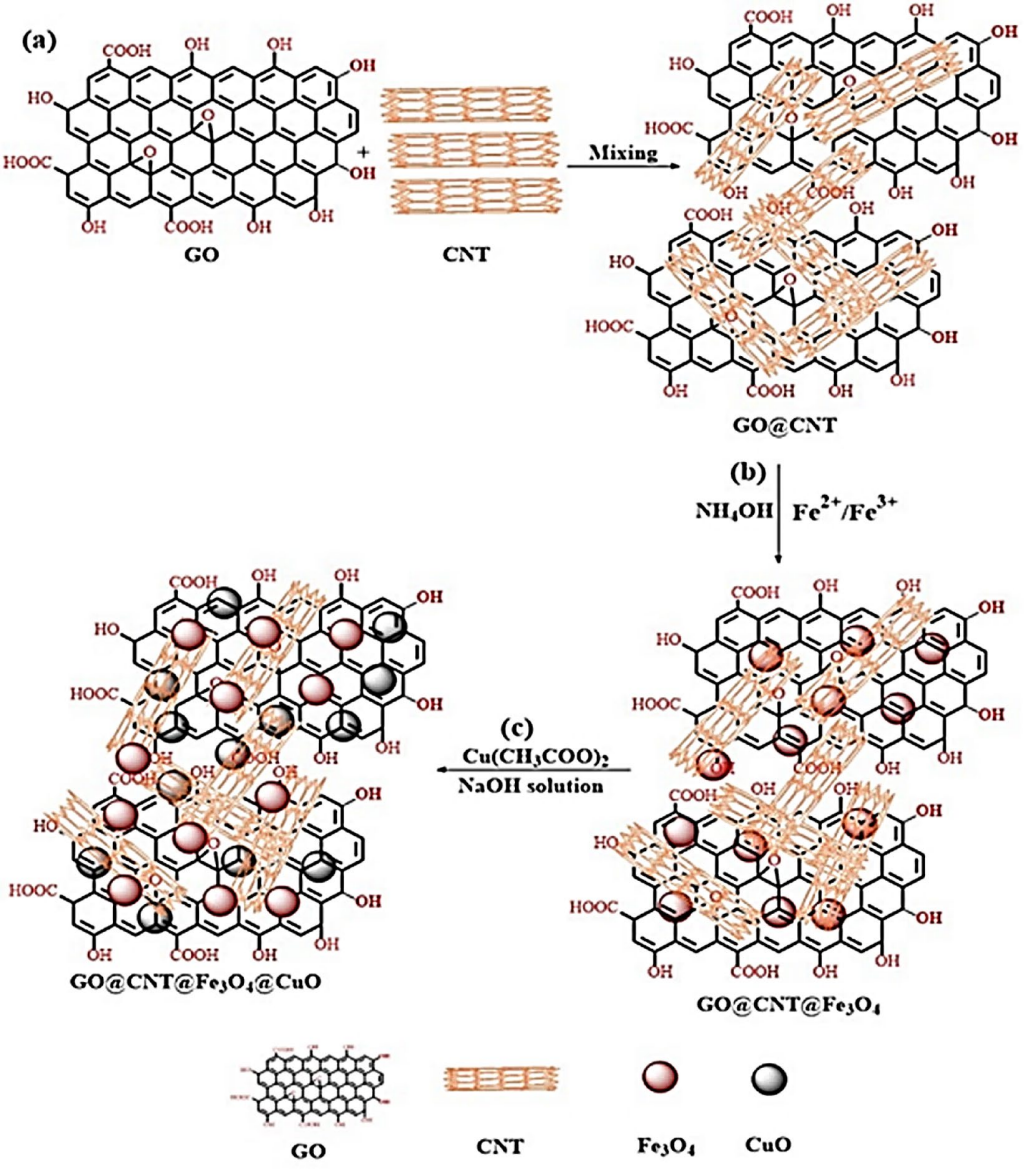
Schematic diagram showing the preparation procedure of GO@CNT@Fe_3_O_4_@CuO core-shell nanohybrid.

The CuO nanoparticles were deposited onto the GO@CNT@Fe_3_O_4_ nanostructure. Figure 1 shows the complete synthesis process for creating the GO@CNT@Fe_3_O_4_@CuO core-shell nanohybrids. In brief, 1.5 g of the GO@CNT@Fe_3_O_4_ nanohybrid was ultrasonically (40 kHz, 300 W, 30 min) dispersed in 400 mL of DI water to form a uniform suspension. Next, 100 mL of DMF was added to the suspension in a three-necked flask while stirring at 1000 rpm. When the GO@CNT@Fe_3_O_4_ suspension reached 85 °C, a 0.2 M Cu(CH_3_COO)_2_ solution was added to start in situ CuO formation on the GO@CNT@Fe_3_O_4_. The mixture was then heated in an oil bath at 90 °C for 1 h. Afterwards, the GO@CNT@Fe_3_O_4_@CuO was separated by centrifugation and washed several times with DI water and methanol. This washed nanostructure was placed in an autoclave at 180 °C for 12 h. Lastly, the GO@CNT@Fe_3_O_4_@CuO was dried using the freeze-drying method. All steps involved in creating the GO@CNT@Fe_3_O_4_@CuO nanohybrid are detailed in Fig. 1.

### Preparation of epoxy-based nanocomposite

**Fig. 2:**
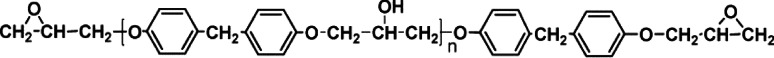
The chemical structure of the Bisphenol-F epoxy resin.

Different nanocomposites, including nanofillers with varying concentrations, were prepared by cross-linking the epoxy resin with an amine cross-linking agent. In this method, the nanofillers were first dispersed in acetone at a predetermined weight ratio, followed by the addition of the resin. The resin-nanofiller mixture was stirred for 30 min to achieve a homogeneous solution. The reaction mixture was then heated to remove the solvent, and the amine crosslinking agent was added at a weight ratio of 1:2 (hardener: resin), stirring at 60 °C for 30 min. The viscous mixture was then transferred into a silicone mold (± 0.1 mm tolerance) and placed in an oven at 60 °C for 1 h followed by RT for 48 h to ensure complete crosslinking without thermal degradation. The nanocomposite was fabricated at various thicknesses (3, 4, and 5 mm). The curing process is essential because it completes the crosslinking reaction, improving the nanocomposite’s mechanical properties and stability. Allowing the material to cure at room temperature for 48 h ensures the polymer’s network structure fully develops, resulting in optimal performance features like greater strength and durability. This final step guarantees that the nanocomposite reaches the required hardness and resilience for its intended uses. All of the nanocomposite compositions are listed in Tables 1and The chemical structure of the Bisphenol-F epoxy resin is shown in Fig. 2.

**Table 1:** Compositions of the various epoxy-based nanocomposites.

Sample code	Resin (g)	Hardener (g)	Nanofiller type	filler wt%	Thickness (mm)
Pure Epoxy	2.0	1.0	0.0	0.0	5.0
E-GO@CNT	2.0	1.0	GO@CNT	5.0	5.0
E-GO@CNT@Fe_3_O_4_	2.0	1.0	GO@CNT@Fe_3_O_4_	5.0	5.0
E-GO@CNT@CuO	2.0	1.0	GO@CNT@CuO	5.0	5.0
E-GO@CNT@Fe_3_O_4_@CuO-5 mm	2.0	1.0	GO@CNT@Fe_3_O_4_@CuO	5.0	5.0
E-GO@CNT@Fe_3_O_4_@CuO-4 mm	2.0	1.0	GO@CNT@Fe_3_O_4_@CuO	5.0	4.0
E-GO@CNT@Fe_3_O_4_@CuO-3 mm	2.0	1.0	GO@CNT@Fe_3_O_4_@CuO	5.0	3.0

^a^The nanofiller content based on the epoxy resin was incorporated into the nanocomposite.

### Characterizations

Fourier transform infrared spectroscopy (FTIR, Bruker, USA) was used to characterize the various functional groups present on the prepared nanofillers. In the analysis, all samples at the same concentration were mixed with KBr and then prepared as transparent pellets using a pressing method. X-ray diffraction (XRD) was employed to investigate the crystalline properties of the samples with a Bruker D8 diffractometer (Germany). A Bruker D8 diffractometer (Germany) was employed to analyze the crystalline properties of the samples. The samples were initially ground into a fine powder to promote uniformity and minimize particle size effects. They were then placed into a sample holder, carefully ensuring a flat and even surface for accurate diffraction outcomes. Before conducting the analysis, the sample holder was precisely inserted into the diffractometer to preserve alignment and measurement accuracy. Additionally, the morphology of the synthesized nanofillers was examined using field-emission scanning electron microscopy (FE-SEM, S4800, Hitachi, Japan) operated at an acceleration voltage of 15 kV. Transmission electron microscopy (TEM, FEI Tecnai G2 F20S-Twin, USA) was employed to analyze the nanostructure morphology in greater detail at an acceleration voltage of 200 kV. This method offered comprehensive information on their size, shape, and arrangement, highlighting essential features that other techniques could not detect. The sample was dispersed in ultrapure ethanol and then cast onto aluminum foil to evaporate the ethanol. Subsequently, the deposited nanostructure was coated with a thin layer of gold to enhance its conductivity properties. Thermal gravimetric analysis (TGA, LENSESSTAPT-1000 calorimeters Germany) was used to evaluate the thermal stability of GO@CNT@Fe_3_O_4_@CuO. TGA characterization was conducted under an argon atmosphere flow (20 mL/min) from 25 °C to 800 °C for the prepared sample. During heating, the temperature increased at a rate of 10 degrees per minute. The EM parameters (ε′, ε″, µ′, µ″) were measured using a vector network analyzer (VNA, AV3629D, Ceyear, China) over a frequency range of 8 to 12.5 GHz. The same parameters were determined with the analyzer across this frequency range. The real permittivity (ε′) reflects the material’s ability to store electrical energy, while the imaginary part (ε″) indicates energy loss as heat. Similarly, the real permeability (µ′) shows the material’s capacity to support a magnetic field, and the imaginary part (µ″) represents magnetic energy dissipated within the material. Understanding these parameters is essential for designing materials with specific electromagnetic properties.

## Results and discussion

To enhance the EM absorption of epoxy nanocomposites, GO@CNT@Fe_3_O_4_@CuO was created through a step-by-step core-shell synthesis process. First, GO nanosheets were produced using the advanced Hummers method and combined with CNTs to form GO@CNT hybrids. Then, magnetic Fe_3_O_4_ nanoparticles were co-precipitated onto this hybrid, resulting in GO@CNT@Fe3O4. To achieve the final GO@CNT@Fe_3_O_4_@CuO core-shell nanofiller, CuO nanoparticles were attached to this ternary hybrid.

### FTIR characterization

**Fig. 3:**
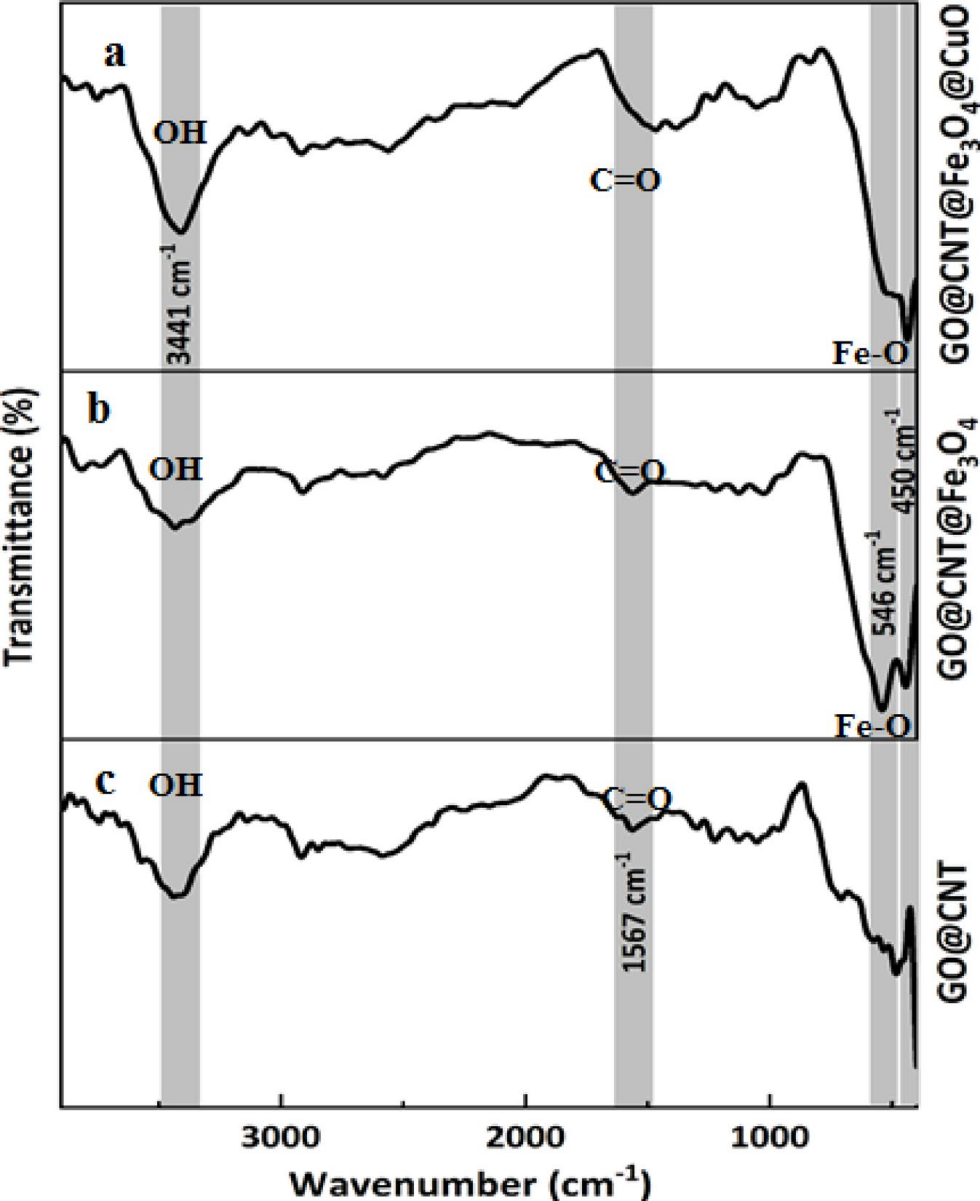
FTIR spectra of the synthesized nanohybrids, such as(a) GO@CNT, (b)GO@CNT@Fe_3_O_4_, and (c)GO@CNT@Fe_3_O_4_@CuO.

FTIR spectroscopy provides essential information about the chemical functional groups on the prepared nanofiller. According to Fig. 3, a broad peak at approximately 3441 cm^−^¹ indicates the stretching vibration of the hydroxyl group (-OH) on the nanomaterial surface and the water molecules absorbed on the nanomaterial^27^. In the GO@CNT sample, peaks appeared at 1624 cm^−^¹ and ~ 1567 cm^−^¹ related to the C = C vibrations in the graphitic area. Additionally, the presence of C-O and C = O functionalities was confirmed by their appearance at 1056 cm^−^¹ and 1726 cm^−^¹ wavenumbers, respectively. These peak observations verified that the GO nanosheets were successfully synthesized and then carefully combined with CNTs. After that, Fe3O4 and CuO nanoparticles formed on the GO@CNT nanostructure. Two new peaks appeared: at 546 cm^−^¹ related to the Fe–O stretching band and at 522 cm^−^¹ linked to the Cu–O vibration in the Fe_3_O_4_ and CuO nanomaterials, respectively^28,29^. All characteristic peaks of the nanomaterials appear in the GO@CNT@Fe_3_O_4_@CuO as the final nanofiller, indicating a successful, step-by-step synthesis that has effectively formed a hybrid of different nanomaterials.

### SEM analysis

**Fig. 4:**
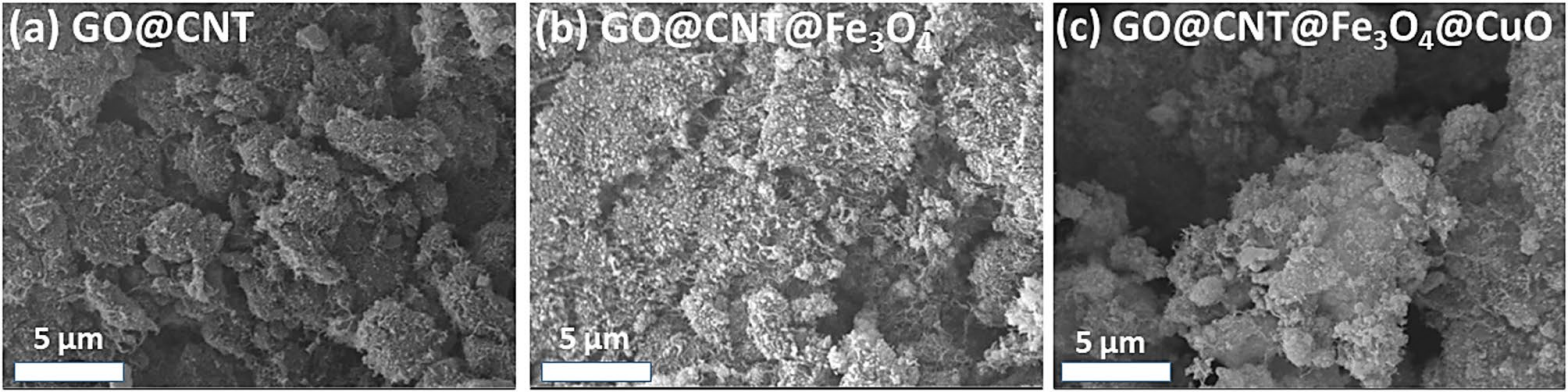
SEM images of (a) GO@CNT, (b) GO@CNT@Fe_3_O_4_, and (c) GO@CNT@Fe_3_O_4_@CuO nanohybrids.

SEM characterization was used to examine the morphology of the prepared nanomaterials. According to Fig. 4a, the GO@CNT shows a combination of both materials: sheet morphology for the GO and tube-like morphology for the CNT. In this case, the CNTs are evenly dispersed on the GO sheets, confirming the successful formation of GO@CNT nanohybrids^30^. According to Fig. 4b, the spherical Fe_3_O_4_ nanoparticles were uniformly formed on the GO@CNT nanohybrid. In the final step, CuO nanoparticles were synthesized on the GO@CNT@Fe_3_O_4_ nanohybrids. Figure 4c shows that the CuO nanoparticles were homogeneously dispersed on the GO@CNT@Fe_3_O_4_ nanohybrids. Comparing Fig. 4b and c, we see that they are very similar because both feature spherical nanoparticles^31,32^. In conclusion, the SEM images show that Fe_3_O_4_ and CuO nanoparticles were successfully nucleated on the GO@CNT nanohybrids.

### TEM characterization

**Fig. 5:**
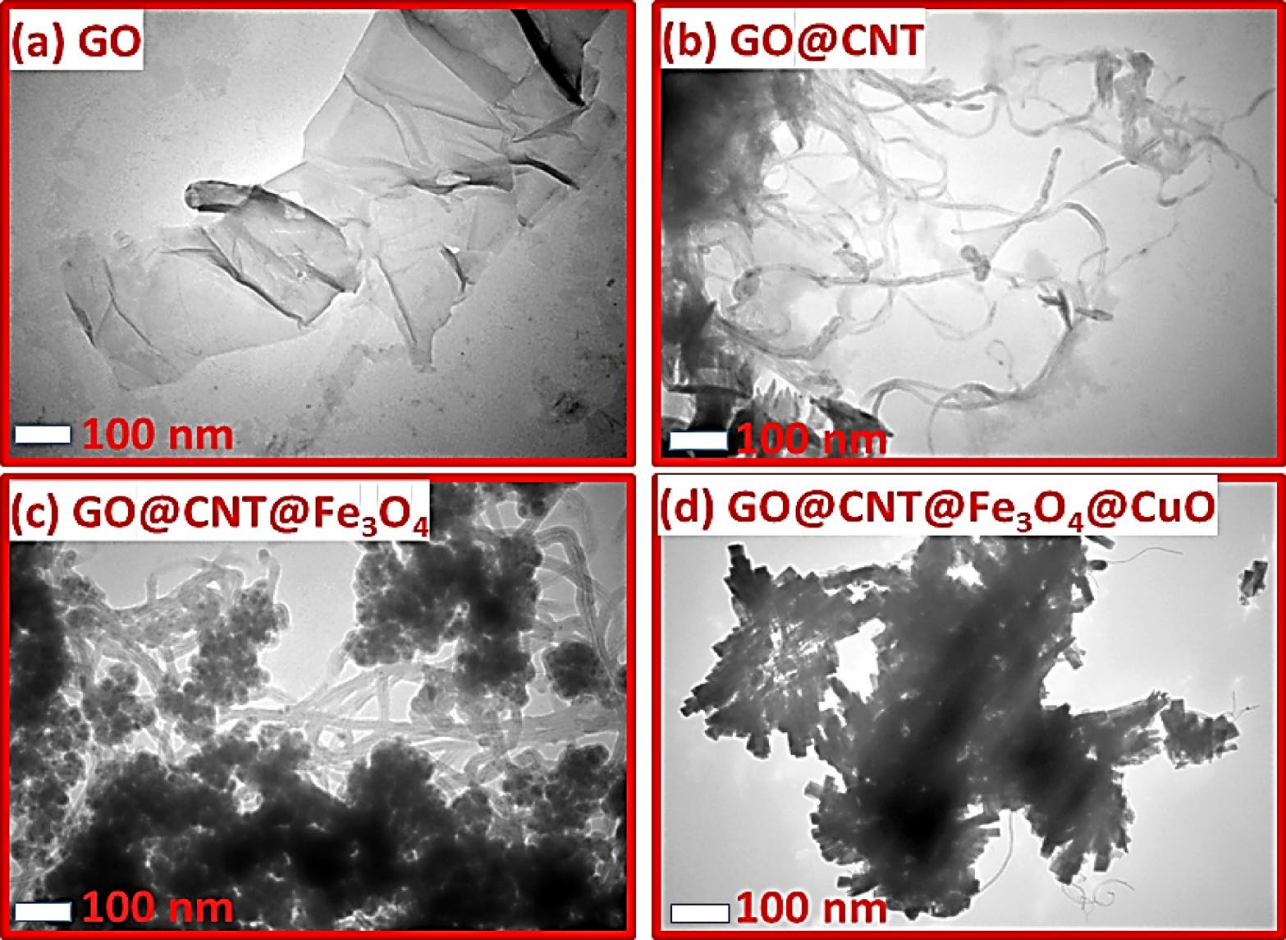
TEM images of the (a) GO, (b) GO@CNT, (c) GO@CNT@Fe_3_O_4_, and (d) GO@CNT@Fe_3_O_4_@CuO.

TEM analysis was used further to study the morphology of the synthesized GO@CNT@Fe_3_O_4_@CuO nanohybrids. The captured TEM images are shown in Fig. 5. Figure 5a confirms that the GO nanosheets have wrinkled sheets with smooth surfaces^33^. When the CNT was grafted on the GO surface, the morphology was significantly altered. A uniform mixture of GO layers and CNTs (30 nm) was observed in this sample^34,35^. Figure 5c shows that the uniform Fe_3_O_4_ nanoparticles (27 nm) form densely on the GO@CNT nanohybrid, without causing agglomeration.

Lastly, the TEM image of the GO@CNT@Fe_3_O_4_@CuO nanohybrid appears darker than the other images. Hence, CuO nanoparticles have been successfully formed without phase separation on Fe3O4 and GO@CNT surfaces.

### Thermal stability properties

**Fig. 6:**
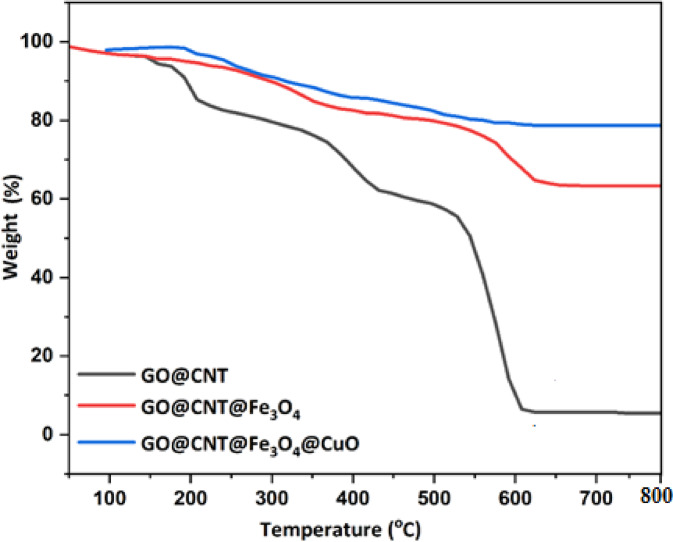
TGA curves of GO@CNT, GO@CNT@Fe_3_O_4_, and GO@CNT@Fe_3_O_4_@CuO nanomaterials.

The TGA method provides valuable insights into the thermal stability of the prepared GO@CNT, GO@CNT@Fe_3_O_4_, and GO@CNT@Fe_3_O_4_@CuO nanohybrids. As shown in Fig. 6, the TGA profiles of these nanostructures display several weight losses. For the GO@CNT sample, the initial weight loss occurs below 100 °C, which is due to the removal of adsorbed water molecules on the GO@CNT surface^9,28^. The second weight loss occurred between 200 °C and 300 °C, corresponding to the release of labile oxygen-containing functional groups, including carboxyl, hydroxyl, and epoxide, on the GO surface^36,37^. The third weight loss for the GO@CNT nanohybrid occurred between 300 °C and 600 °C, indicating the breakdown of more thermostable functionalities on the CNTs and GO surface^38^. The TGA pattern of the GO@CNT@Fe_3_O_4_ changed significantly compared to GO@CNT, which is linked to the presence of Fe_3_O_4_. As shown in the TGA curves, the thermal stability of GO@CNT@Fe_3_O_4_ increased in the 300–600 °C temperature range^34^. Due to the stabilizing nature of Fe3O4 nanoparticles, which act as physical barriers and catalyze selective graphitization while inhibiting excessive charring through oxygen scavenging^11–13^, prevents char formation due to catalytic graphitization. This is validated by reduced weight loss in TGA curves compared to GO@CNT alone. As shown in the TGA curves, the GO@CNT@Fe_3_O_4_@CuO shells and GO@CNT@Fe_3_O_4_ shells display similar thermal behaviors. This sample demonstrates synergistic stabilization properties between Fe_3_O_4_ and CuO nanomaterials from 300 to 800 °C, highlighting their combined stabilization effects (see Table 2).

**Table 2:** Weight loss percentages of samples in different temperature ranges.

Sample	< 100 °C (%)	200–300 °C (%)	300–600 °C (%)	Residue > 600 °C (%)
GO@CNT	3–4	10–12	60–65	5–7
GO@CNT@Fe₃O₄	2–3	6–8	25–30	62–65
GO@CNT@Fe₃O₄@CuO	2–3	5–6	18–22	77–80

### XRD characterization

**Fig. 7:**
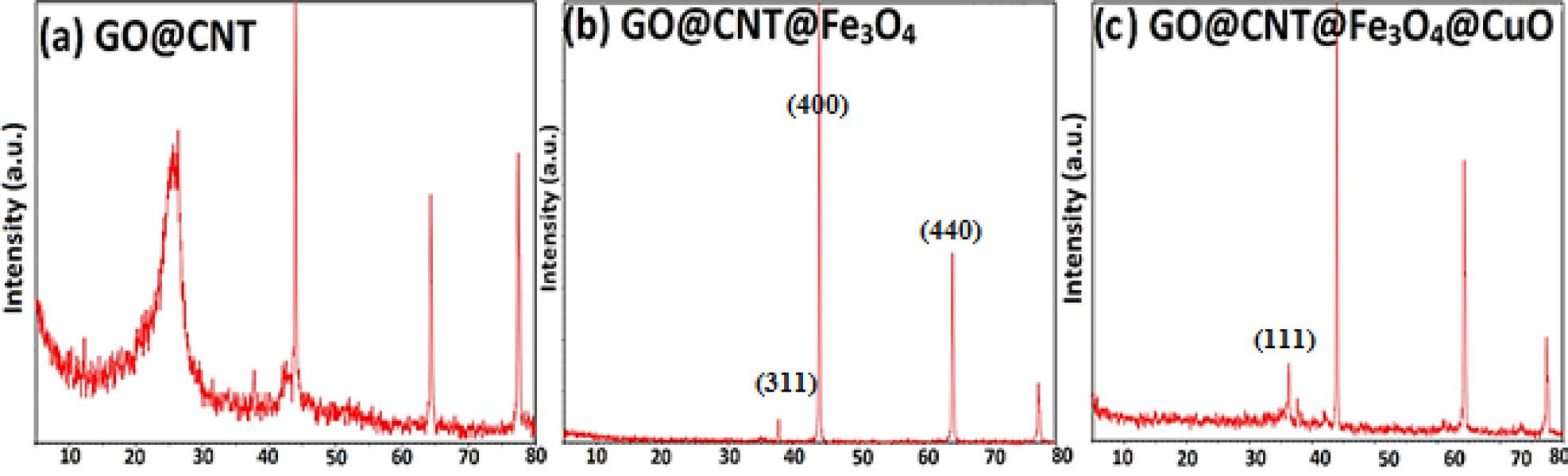
XRD patterns of (a) GO@CNT, (b) GO@CNT@Fe_3_O_4_, and (c) GO@CNT@Fe_3_O_4_@CuO nanomaterials.

XRD analysis was used to characterize the crystalline phase of the step-by-step synthesized nanomaterials, and the recorded XRD patterns are shown in Fig. 7. In the XRD pattern of the GO nanosheets, a broad and weak peak appeared at 2θ ≈ 10–12°, related to the (001) reflection of GO, which indicates an oxidized section of the GO nanosheets^39^. The second peak for the GO@CNT sample appeared at 2θ ≈ 24–26°. This broader peak was attributed to the combined (002) reflections from the unoxidized aromatic regions of the GO and CNTs, confirming the successful hybridization of the GO and CNTs^40^. After anchoring Fe_3_O_4_ on GO@CNT, the XRD pattern changes primarily, and the characteristic peak at around 35.5° (related to the (311) reflection) appears, confirming the crystalline Fe_3_O_4_ nanoparticles (JCPDS 19–0629)^41^. When CuO nanoparticles are incorporated into the GO@CNT@Fe_3_O_4_ to produce GO@CNT@Fe₃O₄@CuO, further new diffraction peaks appear. New peaks at approximately 35.5° for the (11̅1) plane and 38.7° for the (111) plane with ± 0.1° uncertainty, along with reflections from the (002), (200), (202), and other planes (JCPDS 48–1548)^42^.

### Electromagnetic wave absorbing performance of the epoxy-based nanocomposites

To evaluate the EM wave absorption performance of the fabricated nanomaterials, this study calculates their Reflection Loss (RL). Using transmission line theory, RL is determined from the absorber’s complex permittivity and permeability in the following Eqs^43,44^..

1 $${Z}_{in}={Z}_{0}{\left({\mu}_{r}/{\epsilon}_{r}\right)}^{1/2}tanh\left[j\left(2\pi\:fd/c\right){\left({\mu}_{r}{\epsilon}_{r}\right)}^{1/2}\right]$$

2 $$RL\left(dB\right)=20\mathrm{log}\left|\left({Z}_{in}-{Z}_{0}\right)/\left({Z}_{in}+{Z}_{0}\right)\right|$$

Within these equations:

- Z₀ is the impedance of air.
- Z_in_ is the input impedance of the microwave absorber.
- ε_r_ is the complex relative permittivity of the absorber.
- µ_r_ is the complex relative permeability.

Constants c, d, and f represent the speed of light in vacuum, the absorber thickness, and the frequency, respectively. Equations (1) and (2) show that microwave absorption is mainly controlled by the material’s complex relative permittivity (ε_r_ = ε’ - jε’’) and permeability (µ_r_ = µ’ - jµ’’). The real parts (ε’, µ’) indicate the material’s capacity to store electric and magnetic energy, while the imaginary parts (ε’’, µ’’) measure the energy lost as electric and magnetic losses. Polarization mechanisms, including orientational, ionic, electronic, and space charge polarization, influence these dielectric properties.

Within the microwave absorber, the dielectric loss tangent (tan δε) and magnetic loss tangent (tan δµ) measure how efficiently incident electromagnetic energy (electric field energy for tan δε, magnetic field energy for tan δµ) is converted into heat. They are defined as tanδε = ε’’/ε’ and tanδµ = µ’’/µ‘^36^. Effective microwave-absorbing materials require a strong relationship between magnetic and dielectric loss. This study evaluates the EM wave absorption properties of epoxy-based nanocomposites, specifically examining the effects of different nano-fillers and composite thickness.

**Fig. 8:**
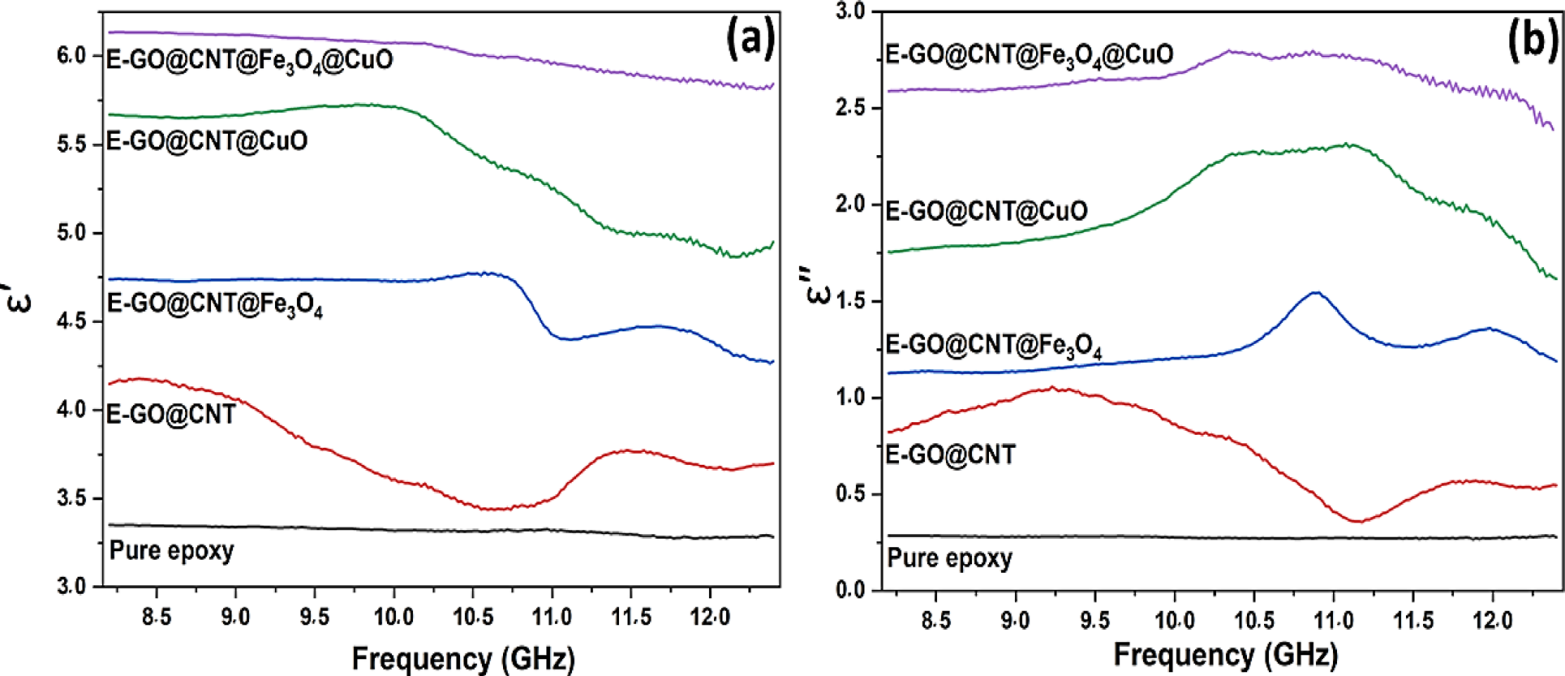
Electromagnetic characterization of absorbers with different nanofillers (GO@CNT, GO@CNT@Fe_3_O_4_, GO@CNT@CuO, and GO@CNT@Fe_3_O_4_@CuO) embedded in epoxy resin across microwave frequencies (8.0–12.5 GHz): (**a**) real part and (**b**) imaginary part of the complex permittivity.

The ε’ and ε’’ trends in the 8–12.5.5 GHz frequency range, using different nanofillers such as GO@CNT, GO@CNT@Fe_3_O_4_, and GO@CNT@Fe_3_O_4_@CuO, are shown in Fig. 7a and b. Based on Fig. 8, the characterization of complex permittivity reveals a consistent enhancement in dielectric characteristics of the epoxy-based nanocomposites as the nanofiller type changes, with the best performance achieved in the GO@CNT@Fe_3_O_4_@CuO-modified nanocomposite. Both the real permittivity (ε’), indicating the material’s ability to store electrical energy, and the imaginary permittivity (ε’’), representing dielectric dissipation loss, demonstrate significant enhancements due to synergistic interactions within the GO@CNT@Fe_3_O_4_@CuO nanofiller structure. In epoxy-based nanocomposites, each component can enhance specific properties to achieve high electromagnetic absorption. For example, GO acts as a high-surface-area substrate with oxygen functionalities. These groups not only help disperse other components within the epoxy matrix but also serve as active sites for dipole polarization^45^. At the same time, GO separates the highly conductive CNT network, preventing agglomeration and promoting the formation of conductive pathways essential for conduction loss (which directly affects ε’’), while also creating numerous GO/CNT/epoxy interfaces that drive interfacial (Maxwell-Wagner-Sillars) polarization^46^. The presence of Fe_3_O_4_ nanoparticles on the GO@CNT nanohybrid (GO@CNT@ Fe_3_O_4_) can cause significant loss mechanisms: eddy currents and natural resonance^47^. Additionally, the electrical conductivity of Fe_3_O_4_ contributes to improving the overall conductivity of the composite^48^. This mitigates the impedance mismatch often seen in highly conductive fillers like CNTs alone, leading to better impedance matching (reflected in a more favorable ε’ profile across the frequency range) and allowing greater EM wave penetration. Additionally, the CNT network forms efficient conductive pathways for minimizing conduction loss and acts as a backbone connecting different phases. The exceptional dielectric performance of the GO@CNT@Fe_3_O_4_@CuO nanocomposite, characterized by the highest and most stable ε’’ values, stems from the synergistic integration of all four components. The co-presence of Fe_3_O_4_ and CuO creates a density of heterogeneous interfaces throughout the epoxy-based nanocomposite. Consequently, the significantly enhanced and well-balanced ε’ and ε’’ profiles of the GO@CNT@ Fe_3_O_4_ @CuO composite directly underpin its superior broadband electromagnetic absorption performance.

**Fig. 9:**
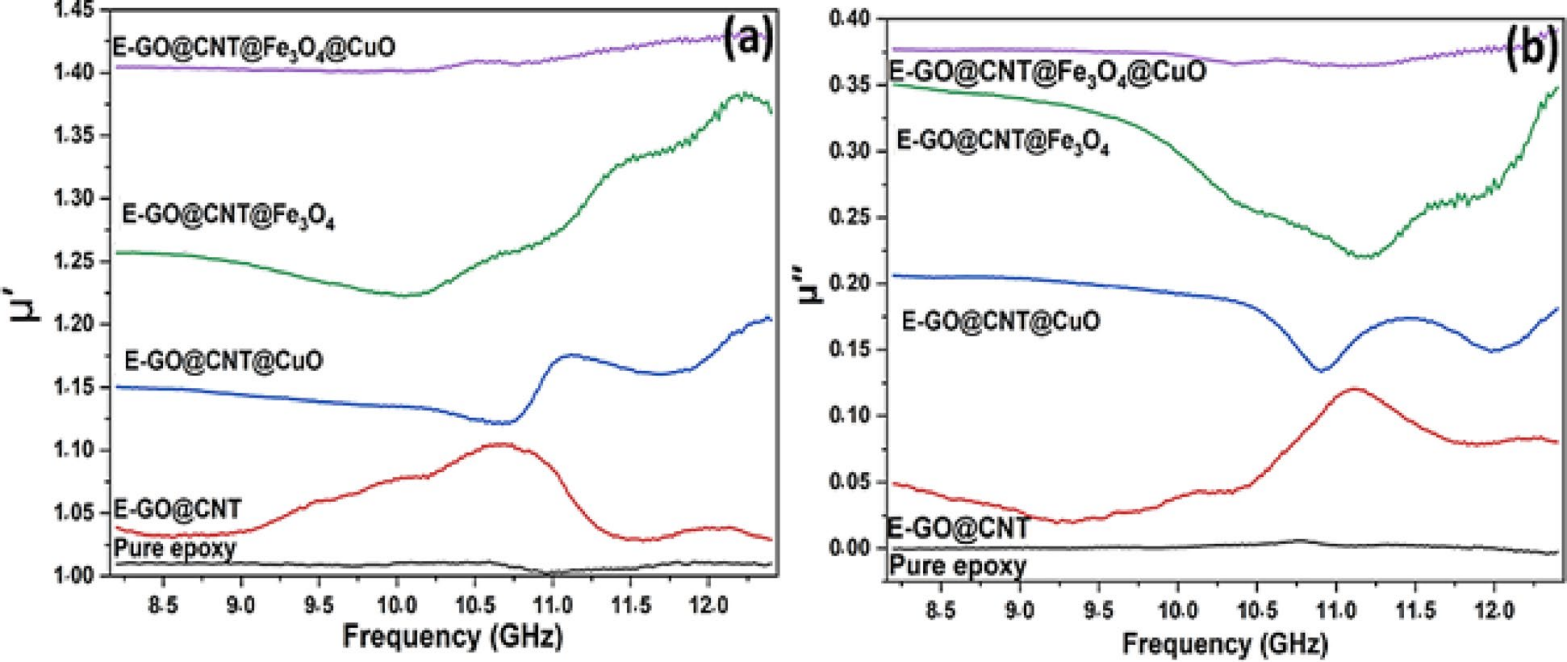
Electromagnetic characterization of absorbers with various nanofillers (GO@CNT, GO@CNT@Fe_3_O_4_, GO@CNT@CuO, and GO@CNT@Fe_3_O_4_@CuO) embedded in epoxy resin across microwave frequencies (8.0–12.5.0.5 GHz): (**a**) real permeability (µ’) and (**b**) imaginary permeability (µ’’).

The complex permeability analysis reveals critical insights into the magnetic contribution to electromagnetic absorption, demonstrating why the GO@CNT@Fe_3_O_4_@CuO/epoxy nanocomposite outperforms others. The real permeability (µ’) represents magnetic energy storage, and the imaginary permeability (µ’’) indicates magnetic dissipation loss. According to Fig. 9, nanocomposites with purely dielectric fillers **–** GO@CNT and GO@CNT@CuO – exhibit µ’ and µ’’ values near unity, confirming their absorption relies solely on dielectric loss mechanisms, as GO, CNTs, and CuO lack intrinsic magnetism. The introduction of Fe_3_O_4_ in GO@CNT@Fe_3_O_4_ fundamentally alters the response, providing significant magnetic loss via natural resonance (oscillation of magnetic moments at GHz frequencies) and potentially eddy currents or exchange resonance^49^. In this ternary composite, GO acts as a high-surface-area scaffold, dispersing Fe₃O₄ nanoparticles and preventing aggregation, which is crucial for maximizing surface area and resonance efficiency. At the same time, the intertwined CNT network may subtly influence magnetic relaxation dynamics. This results in measurable µ’’ values, indicating magnetic energy dissipation^50^. However, the GO@CNT@Fe₃O₄ @CuO nanocomposite achieves the most effective magnetic performance. Although CuO itself is non-magnetic, its integration profoundly enhances the system: it acts as an additional spacer, further refining the dispersion of Fe₃O₄ particles on the GO@CNT scaffold, minimizing aggregation, maximizing active surface area, and optimizing natural resonance efficiency, leading to sustained and well-defined µ’’ values^51^. Furthermore, the complex interfaces formed between Fe₃O₄, CuO, CNT, and GO modify local electromagnetic environments and magnetic relaxation pathways^52,53^. Consequently, the significantly enhanced µ’’ values in GO@CNT@Fe₃O₄ @CuO demonstrate superior magnetic energy dissipation, acting synergistically with its dominant dielectric losses (high ε’’). This powerful combination of strong, complementary attenuation mechanisms (conduction, interfacial polarization, magnetic resonance) and efficient wave entry through balanced impedance matching underpins the exceptional broadband electromagnetic absorption performance of the quaternary GO@CNT@Fe_3_O_4_@CuO-modified epoxy-based nanocomposite.

**Fig. 10:**
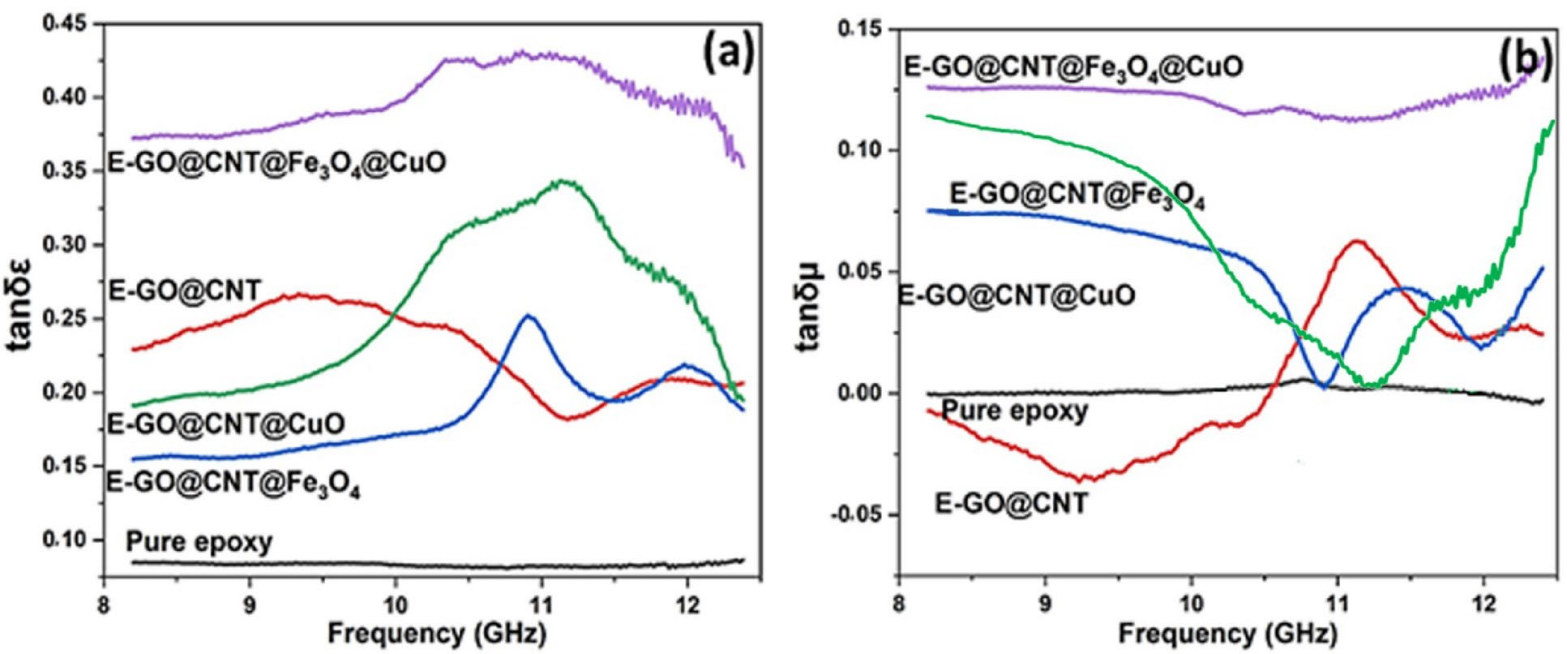
Electromagnetic characterization of absorbers with various nanofillers (GO@CNT, GO@CNT@Fe_3_O_4_, GO@CNT@CuO, and GO@CNT@Fe_3_O_4_@CuO) embedded in epoxy resin across microwave frequencies (8.0–12.5 GHz): (a) dielectric loss tangent (tan δε) and (b) magnetic loss tangent (tan δµ).

Figures 10(a) and 10(b) illustrate the variations in the dielectric loss tangent (tan δε) and magnetic loss tangent (tan δµ) of the epoxy-based nanocomposite with various nanofillers. According to Fig. 10, the data indicate a significant enhancement in the overall electromagnetic wave absorption capacity of the composite as the nanofiller loading rises. This enhancement originates from increased dielectric losses, correlated with a rise in the effective complex permittivity (ε’ and ε’’), likely due to greater electric polarization (including interfacial and dipolar contributions) and conductivity within the epoxy matrix at microwave frequencies. Concurrently, the increase in magnetic losses (tan δµ), associated with the complex permeability (µ’ and µ’’), arises primarily from magnetic hysteresis and domain wall movement. Within the nanofiller, the Fe_3_O_4_ nanoparticles contribute substantially to magnetic loss; their significant magnetic dipole interactions, driven by a high anisotropy field, lead to an increasing lag between the induced magnetization (Ms) and the applied field (Hc) at higher frequencies, thereby elevating magnetic losses^54^. The GO@CNT@Fe_3_O_4_@CuO nanofiller achieves synergistic electromagnetic wave absorption through the distinct roles of its components: where GO provides high dielectric loss, CNT acts as an effective electromagnetic wave absorber, Fe_3_O_4_ nanoparticles serve as the primary magnetic loss component^55^, and the CuO nanoparticles can improve the interfacial polarization. Analysis of the loss tangent data presented in Fig. 10 identifies an optimal GO@CNT@Fe_3_O_4_@CuO within the epoxy matrix for maximizing electromagnetic wave absorption performance.

**Fig. 11:**
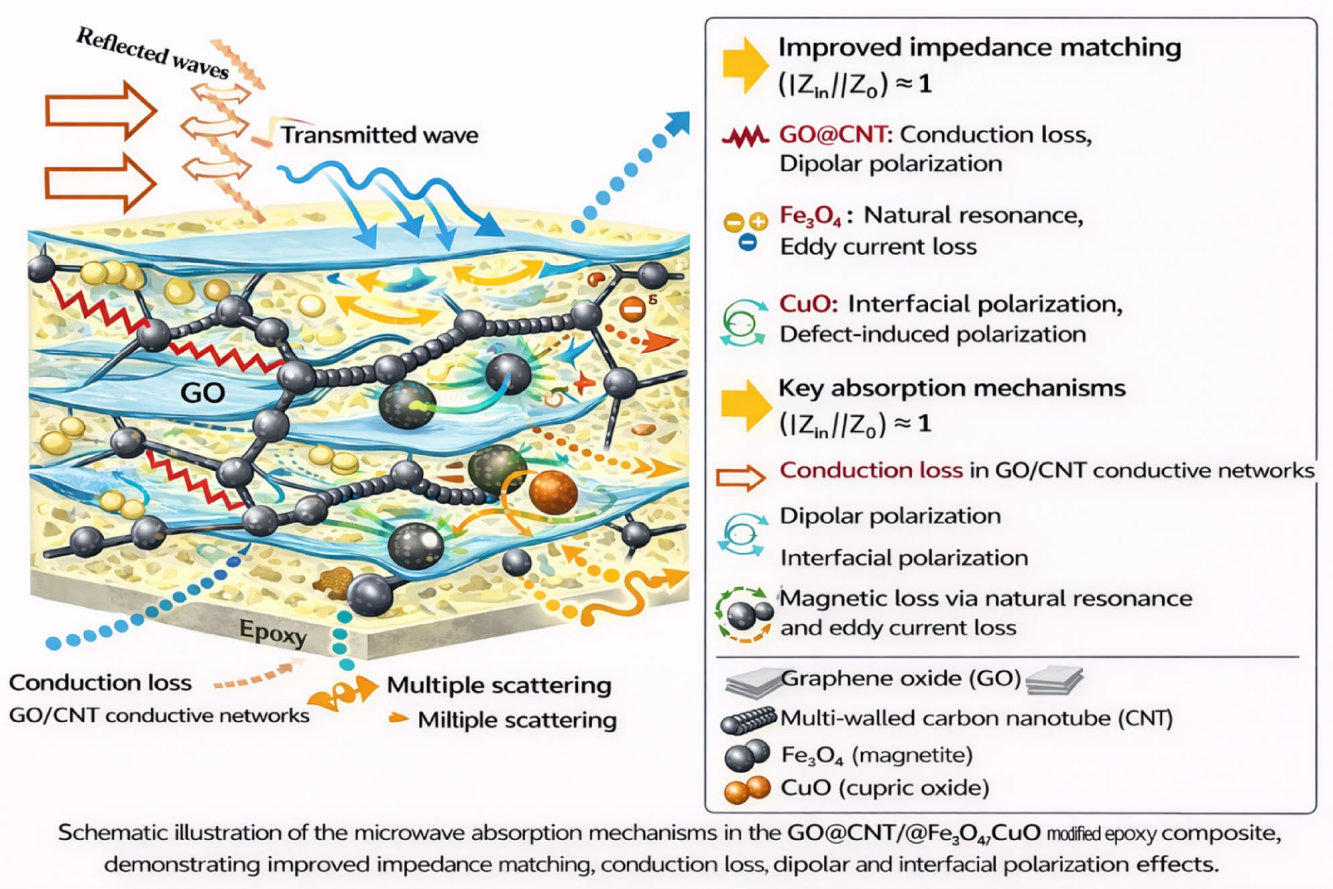
The proposed EM absorbing mechanism of the GO@CNT@Fe_3_O_4_@CuO/epoxy nanocomposite.

Figure 11 illustrates the schematic diagram of the EM wave absorption mechanism in the GO@CNT@Fe_3_O_4_@CuO-modified epoxy nanocomposite. The Fe₃O₄/CuO interface may facilitate interfacial charge transfer and enhanced polarization, potentially due to band alignment effects. The CNT network forms efficient conductive pathways, enabling conduction loss and acting as a backbone connecting different phases.

**Fig. 12:**
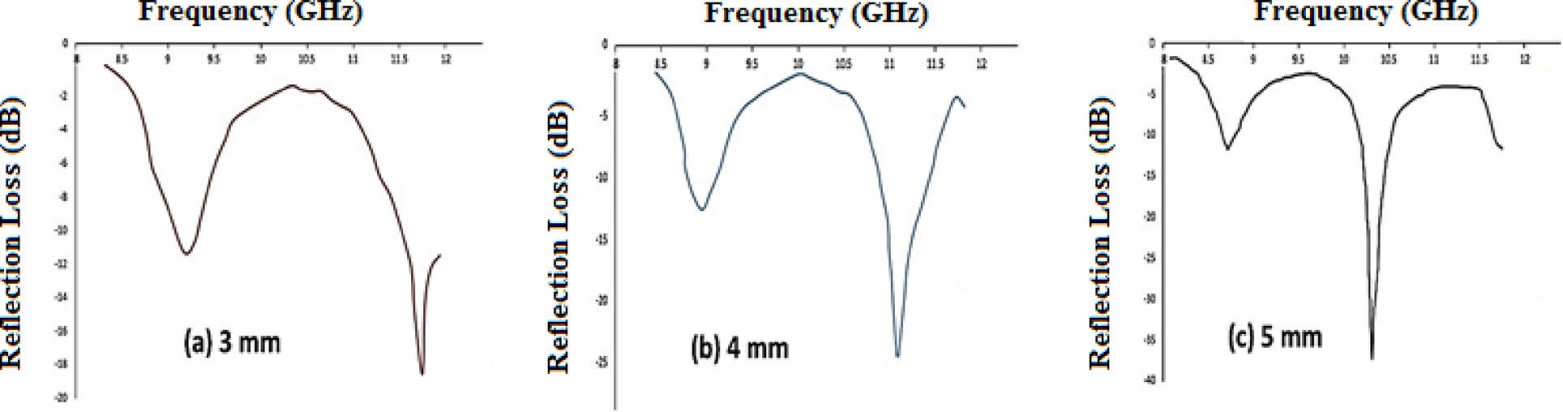
Reflection loss (RL) curves of the GO@CNT@Fe₃O₄@CuO-modified nanocomposite at different thicknesses in the X-band: (**a**) 3 mm, (**b**) 4 mm, and (**c**) 5 mm. Dashed horizontal lines indicate RL = − 10 dB and − 20 dB for reference. The effective absorption bandwidths (EAB, RL ≤ − 10 dB) are 2.0 GHz (10.5–12.5 GHz) at 3 mm, 2.8 GHz (8.4–11.2 GHz) at 4 mm, and 3.2 GHz (9.0–12.2 GHz) at 5 mm.

After selecting the optimal nanofiller type, we systematically studied the EM wave absorption behavior of the GO@CNT@Fe_3_O_4_@CuO-filled epoxy nanocomposites by measuring their reflection loss (RL) across the 8–12.5 GHz (X band) frequency range at different thicknesses (3 mm, 4 mm, and 5 mm). From Fig. 12, the RL variation with frequency indicates that the nanocomposite’s absorption properties heavily depend on its thickness, highlighting the significant role of sample geometry in optimizing EM absorption characteristics. For the thinnest sample, an absorption peak with an absorption bandwidth of approximately 11 to 11.8 GHz was observed. In this sample, RL of about 18.5 dB appeared at a frequency of 11.75 GHz. Increasing the thickness to 4 mm causes a notable shift of the absorption peak to lower frequencies, primarily enhancing the peak’s intensity. The RL value for the nanocomposite with a 4 mm was obtained as −25 dB at 11.2 GHz, indicating good dissipation of incident EM energy. By increasing the thickness of the nanocomposite to 5 mm, the intensity of the absorption peak rises significantly, as shown in Fig. 11, the RL minimum shifts to lower frequencies with increasing thickness, from 11.75 GHz (3 mm) to 10.25 GHz (5 mm), consistent with quarter-wavelength destructive interference theory. The optimal performance is achieved at 5 mm thickness, with a minimum RL of − 37.5 dB at 10.25 GHz and an EAB of 3.2 GHz (9.0–12.2 GHz). This peak shift results from quarter-wavelength attenuation caused by reflected microwave waves from the top and bottom surfaces of the material being out of phase^46,56^. The results demonstrate that the GO@CNT@Fe_3_O_4_@CuO composite effectively absorbs microwaves in the X-band.

**Fig. 13:**
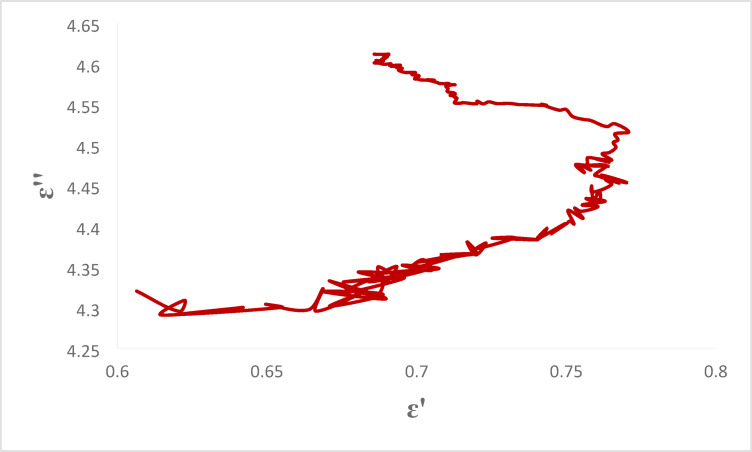
cole-cole plot of the GO@CNT@Fe₃O₄@CuO-modified nanocomposite in the X-band.

Figure 13 presents the Cole–Cole semicircular plot (ε″ vs. ε′) for the E-GO@CNT@Fe₃O₄@CuO nanocomposite in the X-band. The observed non-ideal, flattened arc deviates from the classic Debye model, indicating a broad distribution of dielectric relaxation times. This behavior is attributed to the abundant heterogeneous interfaces within the quaternary architecture—including GO/CNT, CNT/Fe₃O₄, Fe₃O₄/CuO, and nanofiller/epoxy—which generate multiple interfacial polarization processes (Maxwell–Wagner–Sillars effect). The coexistence of these relaxation mechanisms enables effective dielectric loss across a wide frequency range, directly contributing to the broad effective absorption bandwidth (EAB = 3.2 GHz). Such multi-relaxation characteristics have been recently reported in advanced core–shell absorbers, where interfacial engineering between dielectric and magnetic phases plays a decisive role in broadband performance^57,60^. Moreover, the tunable electrostatic interactions between the nanofiller and epoxy matrix further enhance polarization diversity^58,59,61^, consistent with the impedance-matching optimization observed in our system^62,65–70^.

**Table 3:** Comparison of EM absorption performance with recent works.

Material	RL (dB)	f (GHz)	Thickness (mm)	Filler Loading	EAB (GHz)	Ref
MXene/C Aerogel	−58	10.5	2	15 wt%	3.2	^1^
MWCNT/CuO/Fe₃O₄/PANI	−87.4	10.8	2.5	15 wt%	7.6	^17^
RGO/Fe₃O₄	−25	11	5	10 wt%	5	^48^
PANI/Fe₃O₄/CNT	−32.1	10.4	2	20 wt%	1.8	^51^
Co/C (MOF-derived)	−66.5	2–18	1.53	20 wt%	14.3	^63^
PANI/Fe3O4/MWCNTs	−16	8–15	4	20 wt%	8–15	^64^
rGO@CNT@Fe_3_O_4_@ poly (An-co-M)	−17	8–12	5	5 wt%	3.2	^45^
(E-GO@CNT@Fe₃O₄@CuO)	−37.5	10.25	5	5 wt%	3.2	This work

Table 3 provides a comprehensive comparison of the electromagnetic (EM) absorption performance of our GO@CNT@Fe₃O₄@CuO/epoxy nanocomposite with selected state-of-the-art materials, evaluating key metrics such as reflection loss (RL), peak frequency (f), thickness, filler loading, effective absorption bandwidth (EAB), and references. Our material achieves an RL of −37.5 dB at 10.25 GHz with a thickness of 5 mm, filler loading of 5 wt%, and EAB of 3.2 GHz, highlighting its efficiency at low loading due to the quaternary core-shell synergy that balances dielectric and magnetic losses for optimal impedance matching. In contrast, MXene/C Aerogel^1^ offers a deeper RL of −58 dB at 10.5 GHz and similar EAB (3.2 GHz) but requires higher 15 wt% loading and thinner 2 mm structure, potentially limiting mechanical robustness in epoxy matrices. MWCNT/CuO/Fe₃O₄/PANI^17^ excels with − 87.4 dB RL at 10.8 GHz and broad EAB of 7.6 GHz at 2.5 mm, yet its 15 wt% loading increases weight and processing complexity compared to our low-loading design. RGO/Fe₃O₄^48^ shows modest RL (−25 dB at 11 GHz) with wider EAB (5 GHz) at 10 wt% and 5 mm, but lacks the magnetic-semiconducting enhancement for deeper absorption. PANI/Fe₃O₄/CNT^51^ provides RL of −32.1 dB at 10.4 GHz with narrow EAB (1.8 GHz) at lower 2 mm thickness and higher 20 wt% loading, underscoring our superior bandwidth at comparable thickness. Co/C (MOF-derived)^63^ demonstrates exceptional broadband performance (−66.5 dB across 2–18 GHz, EAB 14.3 GHz) at 1.53 mm and 20 wt%, ideal for ultra-thin applications but at the cost of higher filler content. PANI/Fe3O4/MWCNTs^64^ yields shallow RL (−16 dB across 8–15 GHz) at 4 mm and 20 wt%, with full-band EAB but inferior depth. Finally, rGO@CNT@Fe3O4@ poly (An-co-M)^45^ mirrors our loading (5 wt%) and EAB (3.2 GHz) at 5 mm but with weaker RL (−17 dB across 8–12 GHz), emphasizing CuO’s role in enhancing absorption. Overall, our nanocomposite stands out for balanced performance at minimal loading, making it advantageous for lightweight, practical EM shielding in defense and electronics, while addressing limitations in high-loading or narrow-band systems.

## Conclusion

In the current study, we successfully synthesized a series of quaternary core-shell nanostructures, including GO@CNT, GO@CNT@ Fe_3_O₄, GO@CNT@CuO, and GO@CNT@ Fe_3_O₄ @CuO. All of the synthesized nanofillers were characterized using FTIR, SEM, TEM, TGA, and XRD techniques. These characterizations confirmed their thermal stability, chemical structure, and structural integrity. The nanofillers were then incorporated into an epoxy resin to create a novel epoxy-based nanocomposite EM absorber. The EM absorbance performance of all nanocomposites with different nanofillers was evaluated via VNA characterization and compared to that of the neat epoxy resin. Results showed that all modified nanocomposites exhibited better EM absorbance than the unmodified epoxy resin, with the epoxy-based nanocomposite containing GO@CNT@ Fe_3_O₄ @CuO nanofillers identified as the best performer. The high EM absorbance performance of the GO@CNT@ Fe_3_O₄ @CuO nanofillers can be attributed to the synergistic effects of their components: (1) GO nanosheets provide a high surface area substrate for uniform dispersion, (2) CNTs act as conductive networks for conduction loss mechanisms, (3) Fe_3_O₄ nanoparticles help control magnetic loss mechanisms to improve impedance matching, and (4) CuO nanoparticles enhance dielectric loss through improved interfacial effects. Additionally, the GO@CNT@ Fe_3_O₄ @CuO, as a multi-component nanofiller, can form several heterogeneous interfaces, thereby improving interfacial polarization. Although our study shows that the GO@CNT@ Fe_3_O₄ @CuO-modified epoxy nanocomposite is highly effective for high-performance electromagnetic absorption and shielding, it also has limitations. These issues, including thickness dependence, nanofiller dispersion challenges, scalability, and cost, can be addressed through further research and development based on our findings. Future investigations could explore areas such as thin-film engineering, environmentally friendly synthesis methods, and broadband performance optimization. While promising, practical deployment requires addressing challenges in large-scale synthesis, CNT/CuO handling safety, and long-term environmental stability.

## Data Availability

The data that support the findings of this study are available on request from the corresponding author.
